# Disrupting Protein Expression with Peptide Nucleic Acids Reduces Infection by Obligate Intracellular *Rickettsia*

**DOI:** 10.1371/journal.pone.0119283

**Published:** 2015-03-17

**Authors:** Rebecca S. Pelc, Jennifer C. McClure, Simran J. Kaur, Khandra T. Sears, M. Sayeedur Rahman, Shane M. Ceraul

**Affiliations:** Department of Microbiology and Immunology, University of Maryland School of Medicine, Baltimore, Maryland, United States of America; University of Würzburg, GERMANY

## Abstract

Peptide Nucleic Acids (PNAs) are single-stranded synthetic nucleic acids with a pseudopeptide backbone in lieu of the phosphodiester linked sugar and phosphate found in traditional oligos. PNA designed complementary to the bacterial Shine-Dalgarno or start codon regions of mRNA disrupts translation resulting in the transient reduction in protein expression. This study examines the use of PNA technology to interrupt protein expression in obligate intracellular *Rickettsia* sp. Their historically intractable genetic system limits characterization of protein function. We designed PNA targeting mRNA for *rOmpB* from *Rickettsia typhi* and *rickA* from *Rickettsia montanensis*, ubiquitous factors important for infection. Using an *in vitro* translation system and competitive binding assays, we determined that our PNAs bind target regions. Electroporation of *R*. *typhi* and *R*. *montanensis* with PNA specific to *rOmpB* and *rickA*, respectively, reduced the bacteria’s ability to infect host cells. These studies open the possibility of using PNA to suppress protein synthesis in obligate intracellular bacteria.

## Introduction

Organisms in the genus *Rickettsia* are vector-borne Gram-negative α-proteobacteria that cause severe febrile disease in humans. The genus is grouped into Typhus Group (TG) and Spotted Fever Group (SFG) based on antigenicity of outer membrane proteins and the different diseases caused. The obligate intracellular lifestyle of *Rickettsia* species substantially limits the ability of researchers to genetically manipulate them in the laboratory, thus impeding progress in understanding their unique lifestyles and virulence. The use of homologous recombination in *R*. *typhi* [1] and transposon mutagenesis in *R*. *monacensis* and *R*. *prowazekii* [2–4] to express GFP indicate that the barriers to genetic manipulation are not insurmountable. Likewise, the use of the *mariner*-based *Himar1* transposon system enabled researchers to inhibit actin-based motility of *R*. *rickettsii* through disruption of the autotransporter Sca2 and restore a nonlytic plaque phenotype to *R*. *rickettsii spoT* mutants [5,6]. However, these tools are underdeveloped when compared to those available to free-living bacterial systems and present challenges of their own. For instance, screening rickettsial transformants, is constrained by the number of resistance markers available due to the clinical importance of many antibiotics, and by the potential for spontaneous mutants arising following numerous passages [7]. In addition, the process of selecting mutants can take weeks due to poor transformation efficiencies and slow bacterial growth [5,6]. The need for better developed strategies for suppressing protein synthesis in obligate intracellular systems, such as *Rickettsia*, remains in order for improved molecular dissection of host-microbe interactions.

Antisense technologies offer temporary reduction of protein expression through sequence-specific recognition of mRNA. Peptide Nucleic Acids (PNAs) are DNA mimics possessing a pseudopeptide backbone with conventional purine and pyrimidine bases [8]. Antisense PNA molecules complementary to the Shine-Dalgarno or start codon region of mRNA effectively reduce translation [9,10]. PNAs designed complementary to essential components such as ribosomal RNA demonstrate their use as effective antimicrobials in a concentration dependent manner [9], while offering the possbility of using PNA to study essential protein function where a true knockout would preclude recovery of viable mutants. The ability of PNA to limit growth of facultative intracellular bacterium *Brucella suis* [11], implies the feasibility of using this strategy for reducing protein expression in obligate intracellular *Rickettsia* sp.

With this report we aim to provide a proof of principle for the utility of PNA-antisense technology to study *Rickettsia* spp. Based on the efficiency of protein inhibition demonstrated for bacteria from other genera, we hypothesize that PNA will reduce protein expression of RickA and rOmpB resulting in decreased infection of host cells. We targeted *rickA* from non-pathogenic *R*. *montanensis* (SFG) and *rOmpB* from endemic typhus-causing *R*. *typhi* (TG) because of the defined phenotypes associated with their functions. Studies targeting the *R*. *rickettsii* Arp2/3 activator RickA demonstrate its essential role during actin tail polymerization and intercellular spread [12]. Meanwhile, surface expressed rOmpB of *R*. *conorii* is sufficient for bacterial invasion of non-phagocytic mammalian cells [13,14]. Hence, disruption of either RickA or rOmpB expression should limit bacterial load due to their respective roles in establishing infection. Using *in vitro* translation and PNA-RNA hybridization assays, we show that the PNAs hybridize to the complementary upstream region for each target gene. When targeting PNA to either *rickA* in *R*. *montanensis* or *rOmpB* in *R*. *typhi* prior to *in vitro* infection, subsequent burden of infected L929 or Vero cells decreased 88% and 56%, respectively when compared to non-targeting controls at 24 hours. An 80% drop in *R*. *montanensis* burden persisted at 48 hours post-infection. Despite this, we measured no change in adherence to host cells following either *rOmpB* or *rickA* PNA treatments. We detected a 90% reduction in burden *in vivo* following injection of *Dermacenter variabilis* ticks with *rickA* PNA-treated *R*. *montanensis*. Taken together, these data point toward PNA as a viable methodology for studying genes of obligate intracellular species by successfully decreasing protein expression to elicit a phenotype.

## Materials and Methods

### Rickettsiae, host cell culture, and ticks

L929 (Mouse fibroblast, ATCC CCL-1) or Vero76 (African green monkey kidney ATCC CRL-1587) cells were cultured in Dulbecco’s Modified Eagle Medium (DMEM) supplemented with 5% FBS at 34°C and 5% CO_2_. Host cells were inoculated with either *R*. *montanensis* strain M5/6 or *R*. *typhi* strain Wilmington (ATCC VR-144) at 80% confluency. Infected host cells were grown for 5 to 6 days. Rickettsiae were harvested by scraping infected cells into the media and sonicating on ice using a Sonic Dismembranator (Thermo Fisher Scientific Inc., Waltham, MA) for 5 cycles of 7 seconds on with 10 second rests in between. The lysates were centrifuged at 1000x g for 10 minutes to remove large host cell material. The rickettsial suspension was placed over an equal volume of 20% OptiPrep Density Gradient medium (Sigma-Aldrich, St. Louis, MO) in water or SPG buffer (218 mM sucrose, 3.76 mM KH_2_PO_4_, 7.1 mM K_2_HPO_4_, 4.9 mM potassium glutamate) and centrifuged at 10,000x g for 10 minutes. The pellets were washed with 250 mM sucrose, centrifuged at 14,000x g; and MOI 10 determined using the BacLight Live/Dead assay (Life Technologies, Grand Island, NY) or previous estimates. Unfed male and female *D*. *variabilis* ticks were provided by Daniel Sonenshine (Department of Biological Sciences, Old Dominion University). Maintenance of the tick colony was carried out according to protocols approved by the Institutional Animal Care and Use Committee at Old Dominion University.

### PNA Synthesis, labelling, and electroporation

Custom PNA oligomers (Table 1) were synthesized by Bio Synthesis (Lewisville, TX). Oligomers were designed complementary to the predicted Shine Dalgarno region or inclusive of the start codon. A BLASTn analysis was performed for each PNA sequence against the full genomes of each respective bacterium to confirm specificity for the sequence of interest. PNA oligomers were biotinylated using EZ-Link Sulfo-NHS-Biotin, (Thermo Fisher Scientific Inc., Waltham, MA). Labeled PNA was purified using Pierce C18 Spin Columns (Thermo Fisher Scientific Inc.) Biotin-labeled PNA was used to confirm rOmpB PNA binds to the targeted region. Dot blots were used to confirm the successful biotinylation of oligomers. To deliver, 2 μM PNA was mixed with 1 x 10^7^ purified rickettsia in a total volume of 100 μl of 250 mM sucrose in a 0.2 cm gap sterile cuvette on ice. Rickettsiae were then electroporated using a Gene Pulser Xcell Microbial System (Bio-Rad, Hercules, CA) by applying a 5 ms pulse (2500 V, 25 μF, 200 Ω). The PNA-rickettsiae suspension were transferred to a microcentrifuge tube and 400 μl of complete DMEM (5% FBS) medium was added to the electroporated rickettsia for recovery at room temperature for one hour.

**Table 1:** PNA Sequences.

Target Gene	Description	PNA Sequence
***rOmpB***	**Encodes an outer membrane protein sufficient for adherence and invasion of non-phagocytic cells by rickettsiae**	**H-(KFF)**_**3**_ **K-TTT TTG AGC CAT AAT TT-NH**_**2**_
***rickA***	**Encodes a human WASP-like protein with the capacity to activate Arp 2/3 of host cells for intercellular spread**	**H-(KFF)**_**3**_ **K-ACC TAC TAT AAA T-NH**_**2**_
**Non-target-1**	**Scrambled control**	**H-(KFF)**_**3**_ **K-TCT TAG TAT GTA TCT TA-NH**_**2**_
**Non-target-2**	**Off-target control, encodes rifampin resistance**	**H-(KFF)**_**3**_ **K-TAC CAT ATG AAA-NH**_**2**_

### *In vitro* translation

The region starting 100 base pairs upstream of the start codon through 600 base pairs downstream of the *rickA* gene was amplifed using *rickA* forward primer 5’- ATTCGTTCATCTATCTTTTTTTTATTTATC-3’ and reverse primer 5’-TTTTTGTATTTCTTTAAGTTCTTTGACATTAG-3’ and cloned into pEXP5-CT (Life Technologies, Grand Island, NY). Translation was performed using the Expressway Cell-Free *E*. *coli* Expression System (Life Technologies). PNA targeted to *rickA* was added to the reaction to demonstrate PNA specificity for its target sequence as measured by impaired protein production. The CALML3 control vector was included in each reaction to serve as a loading control and to demonstrate PNA specificity. The total volume of each sample was resuspended in LDS loading buffer (Life Technologies) with reducing agent, heated for 10 minutes at 70°C and run on NuPAGE 4–12% Bis-Tris gels. Gels were transferred to PVDF membranes using an iBlot gel transfer device (Life Technologies). Membranes were probed using an anti-His antibody (1:3000) (Thermo Fisher Scientific, Waltham, MA) and developed using SuperSignal West Pico Chemiluminescent Substrate (Thermo Fisher Scientific, Inc.).

### Labeled PNA hybridization

Following biotinylation, PNA labeling was confirmed via dot blot. Briefly, 1 volume 20X SSC buffer was added to biotinylated PNA. Hybond-N+ nylon membrane (GE Healthcare Life Sciences, Pittsburgh, PA) was wet with 10X SSC buffer and spotted with 2 μl of sample and dried. Membranes were wet with denaturing solution (1.5 M NaCl, 0.5 M NaOH) for 5 minutes and transferred to filter paper soaked in neutralizing solution (1.5 M NaCl, 0.5 M Tris HCl, 0.001 M EDTA) for 1 minute. The membrane was dried, UV fixed and developed using Chemiluminescent Nucleic Acid Detection Module (Thermo Fisher Scientific Inc., Waltham, MA) The target *rOmpB* ssRNA molecule 5’-GAAAAAAUUAUGGCUCAAAAACCAAA-3’ was synthesized by Integrated DNA Technologies, Inc (Coralville, IA). Next, 2.0 μl (120 ng) of ssRNA was mixed with either 235 ng of specific or non-targeting control-2 biotinylated PNA, or ratios of unlabeled to labeled *rOmpB* specific PNA. One microliter of RNase Inhibitor (Clontech Laboratories, Inc. Mountain View, CA) and 250 mM Tris (pH 7.2) were added and the total volume was brought to 15 μl using molecular biology grade water. The samples were incubated at 95° C for 10 minutes and left at room temperature to anneal for 30 minutes to 1 hour. The samples were diluted with loading dye supplemented with 0.1% SDS to give the hybridized PNA-RNA an overall negative charge allowing it to run toward the cathode. The samples were run n a 2% TBE agarose gel and transferred to a nylon membrane using an iBlot semi-dry transfer apparatus (Life Technologies, Grand Island, NY). Membranes were crosslinked with UV light and developed using Chemiluminescent Nucleic Acid Detection Module (Thermo Fisher Scientific Inc., Waltham, MA).

### RickA and OmpB protein reduction by titered Western blot

Rickettsiae were purified from L929 cells over an Optiprep cushion as described above. Approximately 5 x 10^6^ of purified *R*. *montanensis* or equal volumes of purified *R*. *typhi* were resuspended in 100 μl of 250 mM sucrose containing 2 μM of PNA targeting *rickA* or *rOmpB*, or non-targeting control-2. The rickettsia-PNA suspension was placed into a pre-chilled 0.2 cm cuvette and electroporated as described above. The rickettsiae were transferred to a microcentrifuge tube and 400 μl of complete DMEM (5% FBS) medium was added to the electroporated rickettsia for recovery at room temperature for one hour. Rickettsia were centrifuged at 17,000x g for 10 minutes, the pellet resuspended in 39.5 μl of water, and two-fold serial dilutions were performed using water as the diluent. LDS sample buffer containing reducing agent was added to each tube containing a dilution for a total volume of 60 μl. The total volume of each sample was boiled for 10 minutes at 90°C, allowed to cool, and separated on a 4–12% Bis-Tris Bolt polyacrylamide gel (Life Technologies). The proteins were transferred to PVDF as described above and probed with anti-Ef-Ts (1:1000, load normalizer) and probed with either anti-RickA (1:2000) or anti-rOmpB (1:250) antibodies overnight at 4° C. Primary antibodies were detected with HRP-labeled donkey anti-rabbit IgG (BioLegend, San Diego, CA) at 1:3000 for 30–45 minutes. Blots were developed with WestPico Chemiluminescent detection substrate (Thermo Fisher Scientific Inc., Waltham, MA). Band densitometry was quantified using GeneTools image analysis software (Syngene USA, Frederick, MD).

### Host cell infection with PNA treated rickettsiae

Following purification, PNA treatment and recovery (described above), *R*. *typhi* and *R*. *montanensis* were added to 1 x 10^4^ L929 or Vero cells in 8-well chamber (Lab-Tek) slides at an MOI of 10. Bacteria were incubated on cells for 24 or 48 hours then washed with 1X PBS to remove nonadherent bacteria and fixed with freshly prepared 3.5% PFA. Extra- and intracellular rickettsiae were stained as previously described [15]. Briefly, extracellular bacteria were probed with either a mouse-anti-*R*. *montanensis* (1:200) or rat-anti-*R*. *typhi* serum (1:500), followed by either a goat-anti-mouse Alexa Fluor 594 or goat-anti-rat Alexa Fluor 594 secondary antibody (1:1000) (Life Technologies, Grand Island, NY). Host cells were permeabilized with 0.1% Triton-X 100 in PBS for 5 minutes at room temperature. Intracellular bacteria were probed as described above using the same primary antibodies but Alexa Fluor 488 secondary antibodies. Samples were mounted under Vectashield with DAPI (Vector Labs, Burlingame, CA) and visualized under oil at a total magnification of 1000x on a Nikon Eclipse E600 microscope. The slides were analyzed using QCapture Pro 5.1 imaging and analysis software. Intracellular rickettsia fluoresced as only green. Percent infection was determined by dividing the number of infected host cells by the total number rickettsiae-associated of cells. Percent association was determined by dividing the number of rickettsiae-associated cells by the total number of counted host cells. Host cells with one or more attached extracellular rickettsiae were considered “rickettsiae-associated.”

### Tick infection with PNA-treated *R*. *montanensis*

Unfed male and female *D*. *variabilis* ticks were injected with 5000–15,000 *rickA* PNA-treated *R*. *montanensis* using pulled glass capillaries attached to a Nanoject II pump (Drummond Scientific, Broomall, PA). Ticks injected with non-targeting control-2 PNA-treated *R*. *montanensis* served as a control. Ticks were injected through the emargination cavity and incubated for 18 hours at 23°C with 95% humidity. Ticks were washed with 3% hydrogen peroxide, sterile water, 70% ethanol in succession, dried and placed in sterile 5 ml conical tubes on ice. Each tick was placed in a drop of AL buffer (DNeasy Blood and Tissue Kit, Qiagen, Valencia, CA) on a specimen slide and cut in two halves using a sterile razor blade. Each tick half and the dissecting buffer was removed to 180 μl of AL buffer. Tick samples were homogenized using a Tissuelyzer (Thermo Fisher Scientific Inc., Waltham, MA) and gDNA isolated according to the manufacturer’s instructions. Equal amounts of gDNA were placed into a qPCR reaction and the rickettsial housekeeping gene, *gltA*, and tick *actin* amplified using Quanta BioSciences SYBR Green I qPCR kit (Gaithersburg, MD) or Stratagene Brilliant SYBR Green QPCR Mastermix (Cedar Creek, TX). Amplification was done using *glta* forward primer 5’-CAGTCCGAATTGCCAGCTCA-3’ and reverse primer 5’- CGGGCCAAAGTGAGGCAATACCCG-3’ and *actin* forward primer 5’- GGAAGGACCTGTACGCCAACAC-3’ and reverse primer 5’- CGCCGATCTTTCATGGTGGAAGG-3’. Rickettsial burden was estimated by normalizing copies of *gltA* to copies of *actin*. Genomic copies were estimated using standard curves for each gene. Briefly, each gene was amplified with gene specific primers, the amplicon extracted from an agarose gel, and serial dilutions of the DNA performed to cover 10^3^–10^10^ copies. Each dilution was amplified using qPCR. A standard curve plotting cycle thresholds versus Log_10_ gene copies was generated for the purpose of estimating bacterial load per tick.

### Statistical analyses

Experiments were repeated at least twice, with individual ticks considered biological replicates. Each treatment was represented by at least ten individual biological replicates collected from all experiments performed. To exclude outlier data points from statistical analyses, interquatrile range outlier tests were performed. Data from each treatment group were subjected to the Shapiro—Wilk test and log transformed if lacking normality. An *F*-test was used to assess equality of variances between groups. Comparison of each treatment with a control was done via a one-tailed *t-test*. All statistical analyses were performed using Microsoft Excel.

## Results

### *rickA* PNA binds the complementary target *in vitro*

**Fig 1:**
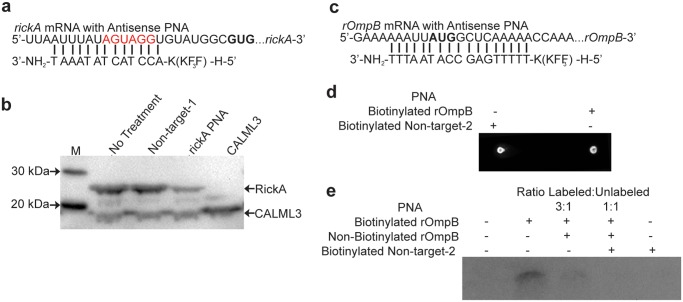
PNA is specific for designed target. A) *rickA* PNA target with putative Shine Dalgarno region in red and start codon in bold B) Western blot of *in vitro* translation assay supplemented with PNA complementary to the cloned region of *rickA*, no PNA, or non-targeting control-1 PNA. *In vitro* translation reactions contained vectors coding both truncated RickA and the control CALML3, or CALML3 alone. C) *rOmpB* PNA target with start codon in bold D) Dot blot confirming biotinylation of *rOmpB* and non-targeting-2 PNA. E) Nylon membrane probed to detect biotinylated PNA-ssRNA pairs following *rOmpB* PNA target RNA denaturation and hybridization to biotinylated PNA, incubated with increasing ratios of unlabeled PNA for competitive binding assay. Incubations with either no PNA or biotinylated non-targeting-2 PNA serve as a control.

PNA complementary to the putative Shine-Dalgarno region of the well-characterized rickettsial gene, *rickA*, was tested for its ability to bind its target and suppress protein production using an *in vitro* translation system. We cloned the gene region starting 100 base pairs upstream of the *rickA* start codon through 600 base pairs downstream to include the sequence complementary to our PNA (Fig. 1A). An *E*. *coli* derived *in vitro* transcription/translation assay was then performed with the reactions treated with either no PNA, non-targeting control-1 PNA, or *rickA* PNA. The control vector coding for human calmodulin-like 3 (CALML3) was added to each reaction to serve as an internal loading control. A separate reaction containing the control vector alone was also performed. The entire volumes of each reaction were processed for Western Blotting to ensure equal loading. Reduced *rickA* production was observed when the *in vitro* translation reaction was supplemented with PNA specific to the gene, as compared with no PNA or non-targeting treatments (Fig. 1B). No change in CALML3 protein level was seen between the treatment groups demonstrating the specifity of the PNA for the *rickA* target sequence and equal loading. However, slightly increased CALML3 expression was seen in the CALML3-only reaction compared with those also including the *rickA* containing vector. This difference is likely due to equal concentrations of reagents being used for the translation of a single protein from a single vector versus the translation of two proteins from two vectors. A ghosted band appears at 25 kDa in all reactions, which may be an artifact of sample preparation, as its presence in the CALML3-only reaction indicates it is not associated with RickA expression. Thus, decreased expression of RickA following treatment with target PNA demonstrates its complementarity to and ability to bind the appropriate nucleotide sequence.

### *rOmpB* PNA is complementary to its target sequence

Attempts to perform PNA treated *in vitro* translation of rOmpB were unsuccessful due to difficulty in cloning the appropriate *rOmpB* region. We chose instead to perform a hybridization assay to demonstrate the complementary binding ability of *rOmpB* PNA (Table 1) to the desired nucleotide sequence (Fig. 1C). To establish *rOmpB-*PNA pairs with the desired sequence, we biotinylated the PNA and hybridized it to a target *rOmpB* ssRNA molecule inclusive of the start codon. Biotinylation of PNA was confirmed via dot blot (Fig. 1D). Equal concentrations of *rOmpB* amplicon were mixed with biotinylated *rOmpB* PNA or non-targeting control-2 PNA, or different ratios of labeled to unlabeled *rOmpB* PNA. This would enable competitive binding to demonstrate specificity. Samples were heated to allow for denaturation then incubated at room temperature to anneal. No PNA hybridization occured when the reaction was supplemented with biotinylated non-targeting control-2 PNA or when the reaction contained no PNA (Fig. 1E). However, hybridization occured when the amplicon was mixed with biotinylated *rOmpB* PNA complementary to the target (Fig. 1E). We saw decreased hybridization of biotinylated PNA following competitive inhibition with increasing concentrations of unlabeled *rOmpB* PNA. This formation of a ssRNA-PNA pairing establishes the ability of our *rOmpB* PNA to bind the desired nucleotide target sequence.

### rOmpB and RickA expression decrease in rickettsiae following PNA treatment

**Fig 2:**
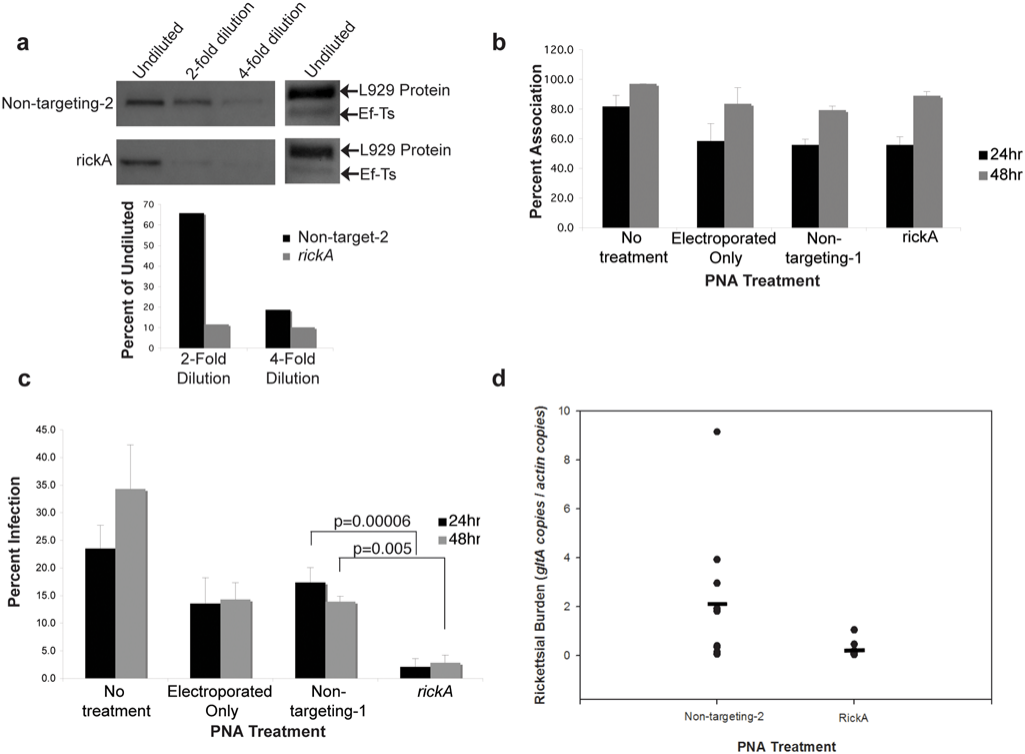
PNA decreases protein production and *in vitro* and *in vivo* infection by SFG *R*. *montanensis*. A) Western blot of Opti-prep purified, serially diluted *R*. *montanensis* treated with *rickA* PNA and quantitative densitometry of the RickA expression as a percent of undiluted RickA. B) Adherence percentages and C) Infection percentages of L929 cells by *R*. *montanensis* treated with PNA designed to *rickA* at 24 and 48 hours. Infection by *rickA* PNA-treated *R*. *montanensis* is reduced 88% (p = 0.00006) at 24 hours and 80% (p = 0.005) at 48-hours post-infection. There is no statistically significant change in adherence with PNA treatment. *In vitro* experiments were repeated twice and performed in duplicate. Error bars represent standard deviation. D) Unfed *D*. *variabilis* adult ticks were injected with *rickA* PNA-treated (n = 11) or non-targeting control PNA-2-treated (n = 10) rickettsia. Rickettsial burden was measured using qPCR. Genomic copies for *gltA* were normalized to genomic copies for *actin*. Closed circles represent individual ticks and the closed horizontal bars represent the mean. Rickettsial burden in ticks infected with *rickA* PNA-treated *R*. *montanensis* is reduced 90% compared to the control (p = 0.004). *In vivo* experiments were repeated twice with 10 biological replicates per treatment.

**Fig 3:**
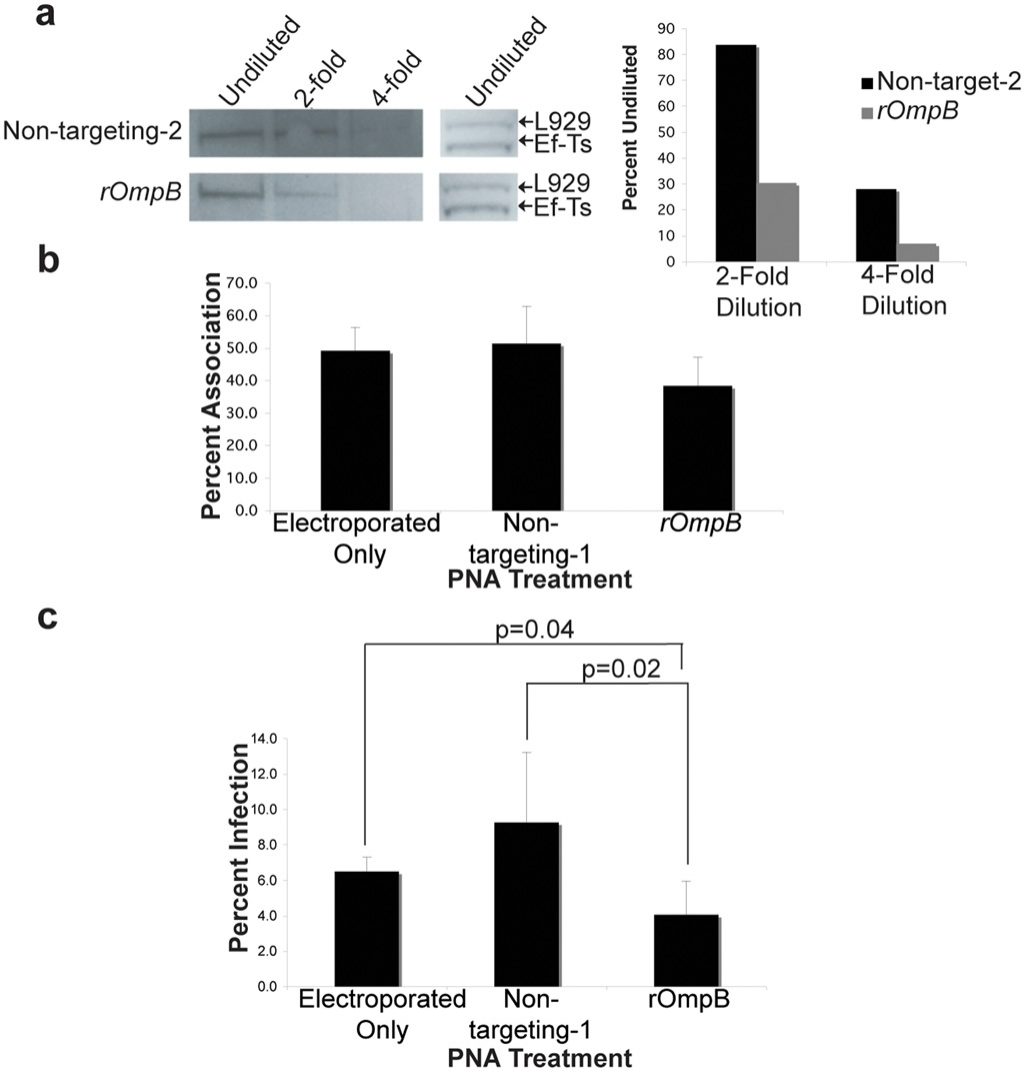
PNA decreases rOmpB protein production and *in vitro* infection by TG *R*. *typhi*. A) Western blot of Opti-prep purified, serially diluted *rOmpB* PNA-treated *R*. *typhi* and quantitative densitometry of the rOmpB expression as a percent of undiluted rOmpB. Adherence percentages of Vero cells by *rOmpB* or non-targetng control PNA-treated *R*. *typhi*. No statistical significance found between treatment groups. C) Infection percentages of Vero cells by *rOmpB* or non-targeting control PNA-treated *R*. *typhi*. Infection by *rOmpB* PNA treated *R*. *typhi* is reduced 56% (p = 0.02) compared to non-targeting control PNA. *In vitro* experiments were repeated twice and performed in duplicate. Error bars represent standard deviation.

Knowing that PNA is specific for its target gene, we evaluated its ability to suppress protein expression in rickettsiae. *R*. *typhi* and *R*. *montanensis* were purified and electroporated with non-targeting control-2 and either *rOmpB* or *rickA* PNA. Following recovery, bacteria were serially diluted, lysed, and protein extracts run on a denaturing gel then transferred to PVDF. The goal of probing a dilution series was to overcome the saturation effect of highly expressed proteins like rOmpB and RickA. Given that PNA-mediated reduction likely affects only a percentage of the whole target protein population, we reasoned that a loss of protein could be measured by a loss of signal at higher dilutions as compared to non-targeting controls. Membranes were probed with antiserum for either rOmpB or RickA and translation elongation factor Ef-Ts, to demonstrate equal loading. We observed a loss of signal earlier in the dilution series in those bacteria treated with PNA specific for *rickA* or *rOmpB* compared with those treated with a non-targeting control-2 (Fig. 2A, Fig. 3A). Meanwhile, undiluted Ef-Ts bands were equivalent. The higher band seen when probing with anti-Ef-Ts serum is a previously documented host protein that cross reacts with our anti-Ef-Ts serum [16,17]. We performed quantitative densitometry graphing the signal of each dilution as a percent of the signal from the undiluted sample. The loss of signal earlier in the dilution series indicates the presence of less rOmpB or RickA, which we interpret as successful PNA knockdown of each protein’s expression respectively.

### Inhibition of Rickettsial entry but not association *in vitro*

RickA and rOmpB are well characterized for their role in intercelluar spread and host cell invasion respectively [12,18–20]. Hence, interruption of these proteins’ expression should reduce rickettsial infection. Purified *R*. *montanensis* were electroporated with either *rickA* or non-targeting-1 PNA, electroporated in the absence PNA, or left untreated. Following recovery, they were incubated with L929 mouse fibroblast cells for 24 or 48 hours. At the respective time points, cells were washed, fixed and stained differentially for internal and external bacteria to allow visual determination of infected or bacterially associated host cells by microscopy as described previously [15]. Bacterial electroporation alone caused a loss of infection compared to no treatment, indicating a loss of viability as a result of the procedure. However, an 88% and 80% drop in infection for *rickA* PNA treated bacteria occurred at 24 and 48 hours respectively, when compared with those electroporated only or treated with non-targeting control-1 PNA (Fig. 2C). Likewise, when we infected Vero cells with *R*. *typhi* treated with either *rOmpB* or non-targeting-1 PNA, we observed a 56% decrease in infection at 24 hours when the bacteria were treated with *rOmpB* PNA versus the non-targeting control (Fig. 3C). Taken together, these data illustrate the capability of using PNA to reduce a particular protein’s expression to in turn impair rickettsiae infection of host cells.

To verify that the loss of host cell infection by *R*. *typhi* and *R*. *montanensis* seen following *rOmpB* and *rickA* PNA treatments was not due to an inability to associate with host cells following PNA treatment, we next measured the ability of bacteria to associate with host cells. We used the aforementioned strategy of differentially staining rickettsiae. Host cells with associated (extracellular) bacteria were counted as a percentage of 100 total host cells. No significant difference in host cell association was seen when *R*. *montanensis* was treated with *rickA* PNA (Fig. 2B) or when *R*. *typhi* was treated with *rOmpB* PNA (Fig. 3B).

### *R*. *montanensis* burden decreases *in vivo* following *rickA* PNA treatment

To evaluate the feasibility of PNA as a strategy for inhibiting protein synthesis *in vivo*, we injected *D*. *variabilis* ticks with *R*. *montanensis* treated with either *rickA* or non-targeting control-2 PNA. Following purification, PNA treatment, and recovery, *R*. *montanensis* were injected into the hoemocel via the emargination cavity of unfed male and female ticks. Ticks were incubated overnight, dissected, and burden assessed using qPCR comparing relative numbers of rickettsial and tick housekeeping genes. Rickettsial burden in the ticks decreased 90% following infection by *rickA* PNA-treated *R*. *montanensis* as compared to non-targeting control-2-treated rickettsia (Fig. 2D). This decrease in bacterial load complements the *in vitro* infection data and establishes PNA as an approach for successful inhibition of rickettsial protein expression leading to an *in vivo* phenotype.

## Discussion

The use of PNA to reduce protein expression and subsequently impair bacterial growth has been investigated for both Gram-negative and Gram-positive bacteria [9,21]. PNA designed complementary to the ribsomal binding sites or inclusive of the start codon of *rickA* and *rOmpB* demonstrated the ability of this anti-sense technology to inhibit gene function essential to infection by rickettsiae. Subsequently, an overall decrease in the amount of RickA or rOmpB, as determined by immunoblotting serially diluted bacteria, is seen in PNA-treated *R*. *montanensis* or *R*. *typhi*, respectively. When treated with PNA specific to *rOmpB*, *R*. *typhi* infection of Vero cells decreased, and when treated with PNA complementary to *rickA*, *R*. *montanensis* infection of L929 cells decreases. Yet, no decrease in the percentage of bacterially associated cells took place with either *rickA* or *rOmpB* PNA treatments compared to controls, suggesting a role for other adhesin factors in the rickettsial adherence to host cells. Reduction in bacterial burden also occured in the tick vector *D*. *variabilis* following infection with *rickA* PNA-treated *R*. *montanensis*.

Traditionally, delivery of PNA occurs by linking the oligo to a basic stretch of amino acids, which increases uptake by free-living bacteria, and adding it to broth culture. While all our PNAs, experimental and non-targeting controls, were conjugated to the cell penetrating peptide (CPP), KFFKFFKFFK, [22] concern regarding the viability of rickettsiae if left extracellular for an extended period of time and the efficacy of getting PNA through both host and bacterial membranes led us to examine electroporation of purified *Rickettsia* as a delivery method. Electroporation is a tested method of delivering nucleic acids to rickettsial species [2–4]. Additionally, electroporation of *Salmonella typhimurium* infected macrophages with CPP-PNA targeting *rpoD* reduced the bacterial load more than those infected macrophages receiving no electroporation, implying it is a feasible method of PNA delivery [23]. While electroporation of purified *Rickettsia* to deliver PNA did cause a loss of bacterial viability (Fig. 2C), the remaining bacteria established productive infection in our control samples, demonstrating the utility of electroporation as a PNA delivery method. We argue that the limitation of infection *in vitro* and *in vivo* is due specifically to the PNA and not the CPP as both our non-targeting controls and experimental PNAs contain the CPP with no deleterious effects. Futhermore, previous work reports that adding CPP-PNA to host cell macrophages causes no detectable toxicity of the cell [11], suggesting that any drop in bacterial load is not due to PNA-induced host cell toxicity.

The reduction in infection seen following PNA treatment echos previous studies investigating the function of *rickA* and *rOmpB* from other rickettsial species. *R*. *rickettsii* Iowa stain, which is defective at cleaving rOmpB, displayed reduced virulence and plaque formation [19]. Likewise, inhibiting Ku70, the mammalian receptor of rOmpB, by antibody blocking or siRNA treatment reduced invasion by *R*. *conorii*, with no effect on bacterial adherence to host cells [20]. Though well characterized for its polymerization of host actin [12,18], recent reports further implicate RickA in invasion. Fewer infected A549 cells per infectious focus were calculated for *rickA*:*tn* mutant when compared with wild type *R*. *parkeri* 24-hours post-infection [24]. Additionally, *R*. *bellii* overexpressing *rickA* from *R*. *monacensis*, adhere to Vero cells in greater numbers at early time points (2–4 hours-post-infection), implying that *rickA* may have a role in the establishment of early infection, as adherence is required for invasion by the bacteria [25]. At 24-hours post-infection we saw less infection but not adherence by *R*. *montanensis* treated with *rickA* PNA, an identical phenotype to what we see when *R*. *typhi* is treated with *rOmpB* PNA. The trend continued with *rickA* PNA treatments at 48-hours post infection. We opted to not take *rOmpB* PNA-treated *R*. *typhi* samples at 48-hours because of the characterization of rOmpB as an invasion factor, presuming that a phenotype associated with reduced rOmpB would be best measured at earlier time points. Conversely, RickA is primarily known as an actin nucleator which could be important at later time points for intercellular spread. Yet, when *D*. *variabilis* tissues are treated with an Arp2/3 inhibitor prior to *R*. *montanensis* infection, burden is reduced, implying a role for actin polymerization in host cell invasion [26]. In addition, our observation that PNA-mediated suppression of RickA expression reduced the ability of *R*. *montanensis* to infect host cells and ticks argues that RickA plays a role during invasion. Consistent with this, our comparable infection and adherence data between *R*. *typhi* treated with *rOmpB* PNA and *R*. *montanensis* treated with *rickA* PNA further suggests that RickA, like rOmpB, may be involved in the host cell invasion process in addition to its well documented role in actin polymerization.

Off target effects are a concern when working with antisense oligos to interrupt gene function. In *E*. *coli*, two base mutations of the target site reduced PNA-mediated inhibition, while six base mutations virtually eliminated it [9]. Six base pair mismatches in either PNA or its target sequence ablated PNA inhibition in *Staphylococcus aureus* [21], further demonstrating the high specificity of PNA for its target sequence. When designing PNA oligos, we blasted the PNA sequences to ensure they aligned with only our target sequences, with our *rickA* PNA designed complementary to the putative Shine-Dalgarno region and the *rOmpB* PNA designed to include the start codon (Fig. 1A, 1C). Our *rickA* PNA inhibits RickA production but not CALML3 in an *in vitro* translation reaction. Additionally, we use two different non-targeting controls (Table 1) in our experiments. Non-targeting-1 was used for *in vitro* translation as well as cell culture infections, while Non-targeting-2 was used for ssRNA hybridization, *in vivo* protein suppression and tick infections. Non-targeting-1 is a scrambled control while Non-targeting-2 is designed for a rifampin resistance gene that our bacteria do not possess. Experimental PNAs, *rOmpB* and *rickA*, produced equivalent expression interruption results regardless of the control being used for comparison. Taken together these data point to the specificity of the technology for a target.

Our treatment of rickettsiae with 2 μM PNA to inhibit protein production is comparable to concentrations used in other systems. GFP expression from GFP-expressing *S*. *aureus* is reduced 30% following treatment with 2 μM PNA [21]. Interestingly, reduction in LacZ expression by *E*. *coli* is recorded following treatment with PNA at nanomolar concentrations [22]. Moreover, a positive correlation between PNA treatment and protein reduction exists in each system [21,22]. These observations suggest that further optimization is needed to establish the PNA dose requirements for the suppression of less ubiquitous targets and to determine the growth inhibitory concentrations for each system studied.

Our ability to recapitulate phenotypes reported by previous studies using PNA, illustrates its potential as a genetic tool in rickettsiae. While advances in the use of transposon delivery [5,6,27] and shuttle vectors [28] have increased the breadth of questions that can be asked about rickettsiae, tools to study obligate intracellular bacteria lag behind those available to study free-living bacteria. The field needs additional tools of genetic manipulation to better scrutinize the lifestyle and virulence of obligate intracellular bacteria, and allow molecular Koch’s postulates to be fulfilled. Our ability to inhibit protein expression in non-pathogenic Spotted Fever Group (SFG) *R*. *montanensis* and pathogenic Typhus Group (TG) *R*. *typhi* indicates the broad spectrum of uses for this technology. We propose that PNA could be utilized by a multitude of intracellular systems as a method for determining essential functions of genes to be targeted in the development of drugs and vaccines. PNA offers an advantage in that protein expression reduction is achieved in one day, no antibiotic selection is required, and partial reduction can be achieved allowing study of essential genes, where investigations of a phenotype imposed by a true knockout would be impossible due to low bacterial viability. For instance, PNA-mediated inhibition of essential genes involved with DNA replication, fatty acid synthesis, and RNA synthesis was successful in facultative intracellular *B*. *suis*[11]. These data also open the possibility of developing other anti-sense methods, such as transforming bacteria with vectors coding for an anti-sense transcript or supplementing host cell culture media with PNA molecules for delivery.

Current methods of delivery to free-living bacteria involve suplementation of culture media with PNA. Electroporation allows synchronization of PNA delivery to the entire bacterial population, increasing the effectiveness of PNA. Furthermore, studies that target proteins with transient expression can be conducted with greater accuracy and precision due to the synchronized method of PNA delivery. It was recently reported that electroporation enhances the availibility of CPP-PNA to *S*. *typhimurium*, following electroporation of infected macrophages compared to CPP-PNA added without electroporation [23]. Given these findings, it is possible that electroporation can reduce the costs of PNA synthesis for use in antisense studies by requiring no CPP and allowing lower concentrations to be used for experiments, but further optimization is required.

Many obligate intracellular bacteria cause serious morbidity and mortality in humans and lack effective vaccines. By targeting rickettsial genes of interest for the suppression of protein synthesis resulting in a gene specific phenotype, PNA offers promise as an additional tool for studying virulence and the intracellular lifestyle of these bacteria. Expanded use of this methodology to temporarily knock down protein expression for genes of interest may also allow the study of essential gene function in obligate intracellular bacteria.
